# A framework for FAIR robotic datasets

**DOI:** 10.1038/s41597-023-02495-3

**Published:** 2023-09-13

**Authors:** Corrado Motta, Simona Aracri, Roberta Ferretti, Marco Bibuli, Gabriele Bruzzone, Massimo Caccia, Angelo Odetti, Fausto Ferreira, Francesca de Pascalis

**Affiliations:** 1 Institute of Marine Engineering (INM), National Research Council of Italy (CNR), Department of Engineering, ICT and Technology for Energy and Transport (DIITET), Genoa, 16149 Italy; 2 Arcadia SIT, Vigevano, 27029 Italy; 3 University of Zagreb, Faculty of Electrical Engineering and Computing, Zagreb, 10000 Croatia; 4 Institute of Marine Sciences (ISMAR), National Research Council of Italy (CNR), Department of Earth System Sciences and Environmental Technologies (DSSTTA), Venice, 30122 Italy

**Keywords:** Ocean sciences, Physical oceanography

## Abstract

It is essential to publish and make available environmental data gathered by emerging robotic platforms to contribute to the Global Ocean Observing System (GOOS), supported by the United Nations - Decade of Ocean Science for Sustainable Development (2021–2030). The transparency of these unique observational datasets needs to be supported by the corresponding robotic records. The data describing the observational platform behaviour and its performance are necessary to validate the environmental data and repeat consistently the in-situ robotic deployment. The Free and Open Source Software (FOSS), proposed in this manuscript, describes how, using the established approach in Earth Sciences, the data characterising marine robotic missions can be formatted and shared following the FAIR (Findable, Accessible, Interoperable, Reusable) principles. The manuscript is a step-by-step guide to render marine robotic telemetry FAIR and publishable. State-of-the-art protocols for metadata and data formatting are proposed, applied and integrated automatically using Jupyter Notebooks to maximise visibility and ease of use. The method outlined here aims to be a first fundamental step towards FAIR interdisciplinary observational science.

## Introduction

The rise of cutting-edge robotic platforms^1–3^ in the context of the Global Ocean Observing System (GOOS)^4^ is rapidly feeding a new generation of data. Both the environmental and the robotic data need a rigorous treatment^5^ capable to align marine robotics data with the long tradition of observational oceanography. FAIR - Findability, Accessibility, Interoperability, and Reusability - principles have to be the steering factors when handling these coupled datasets^6^.

They are, in fact, fundamental to cement data conduit to render scientific studies scrutable and scientific data repeatable and declinable. By following FAIR principles, the gathered data can be used in multiple fields of science not necessarily only within the collecting community, which is more likely to share the same vocabulary and background knowledge. Rendering a dataset FAIR encompasses, among other things, establishing a set of discovery metadata, i.e., descriptive information. Particular data centers/observing systems/research institutes provide tailored guidelines for data publication in their portals. For example, Earth Sciences data follow the NASA Global Change Master Directory - Directory Interchange Format (GCMD DIF)^7^; Arctic data can follow Svalbard Integrated Arctic Earth Observing System (SIOS)^8–10^ guidelines. GCMD DIF and SIOS encompass the more general standards, ISO 19115^11^, but they also integrate requirements that shape data treatment in Earth Sciences. However, in many other contexts, such guidelines are not provided and the datasets are published without sufficient descriptive metadata. In marine robotics, often data are collected during field expeditions and published as raw telemetry. While there are some efforts in place to improve data standardisation^12^, mostly these are either for industrial applications, such as Remotely Operated Vehicles (ROVs)^13–16^, or military-originated^17^, in other cases these attempts only address the construction of marine robots, but not their collected data^18^. The result is a lack of scrutiny when it comes to metadata and metadata standards as FAIRness enablers.

On the semantic level, the nomenclature used for metadata and for the description of variables and their attributes has to comply with a controlled vocabulary. For Earth Sciences, this is described in the Climate and Forecast (CF) Metadata Convention^19^, designed to promote the processing and sharing of files created using the NetCDF (Network Common Data Form) software libraries and machine-independent data formats^20,21^. A shared/controlled vocabulary, for a given discipline, ideally contains the standard names of all the variables that can be stored in a FAIR dataset. There are a few efforts such as Marine Regions^22^ for what concerns georeferencing of marine areas (e.g. for trials) or the vocabularies defined in the NERC Vocabulary Server^23^, for instance for ARGO floats^24^. Environmental and robotic variables are constantly evolving, hence a shared vocabulary is a living entity, continuously developing^25,26^. To the best of our knowledge, such vocabulary does not exist in the field of marine robotics, hence FAIRness cannot be fulfilled in the current state. We are working on a controlled vocabulary dedicated to the robotic variables, which at the moment is not present in literature, capable of maintaining the robotic and environmental dichotomy. For the data collected by marine vehicles, the vocabulary is based upon the Fossen nomenclature^27^, which is commonly consulted by robotic scientists, for instance, when developing the control algorithm of a marine robot. As far as the environmental data are concerned, we base our controlled vocabulary on the Climate and Forecast Convention, which encompasses both the specific data unit of thought and the metadata singular concepts. In fact, fundamental aspects such as interoperability and reusability demand domain-specific standards, as for example the Climate and Forecast Convention. This paper wishes to lay the foundations of a data framework capable of including dynamically new concepts and their corollary entities. Ultimately, FAIR datasets need to be identified by a unique identifier, i.e. a DOI (Digital Object Identifier). An identifier associated uniquely to an object (data, article, abstract) allows the community to keep track of a specific product^28^. Moreover, given the importance of instruments and associated metadata for the assessment of data quality and data reuse, a globally unique, persistent and resolvable identification of instruments is crucial. To this end, the Research Data Alliance (RDA) Working Group (WG) Persistent Identification of Instruments (PIDINST) explored a community-driven solution for globally unambiguous and persistent identification of operational scientific instruments^29^.

In this paper, we present a Free and Open Source Software (FOSS) to render marine robotics datasets FAIR-compliant. The method can be automated to generate a FAIR datasets right after field missions. Achieving complete FAIR compliance using our framework is an iterative and incremental process that we have begun to design starting from the management of metadata, a fundamental aspect in the context of the FAIR principles. In particular, we suggest a minimum set of descriptive metadata for the coupled datasets of robotic and environmental data, in order to guarantee the principle of findability and accessibility. Furthermore, we define use metadata for the variables (attributes) to ensure the reusability of the datasets. Finally, for the sake of interoperability, we propose standard names for the robotic variables that do not belong to any controlled vocabulary, by following the most used terminology in the robotic domain. The method comes with its software implementation, which is also described in the paper and available on GitHub^30^. A practical example of the applicability of the method on real data acquired during field tests is also available on Zenodo repository^31^.

## Results

**Fig. 1:**
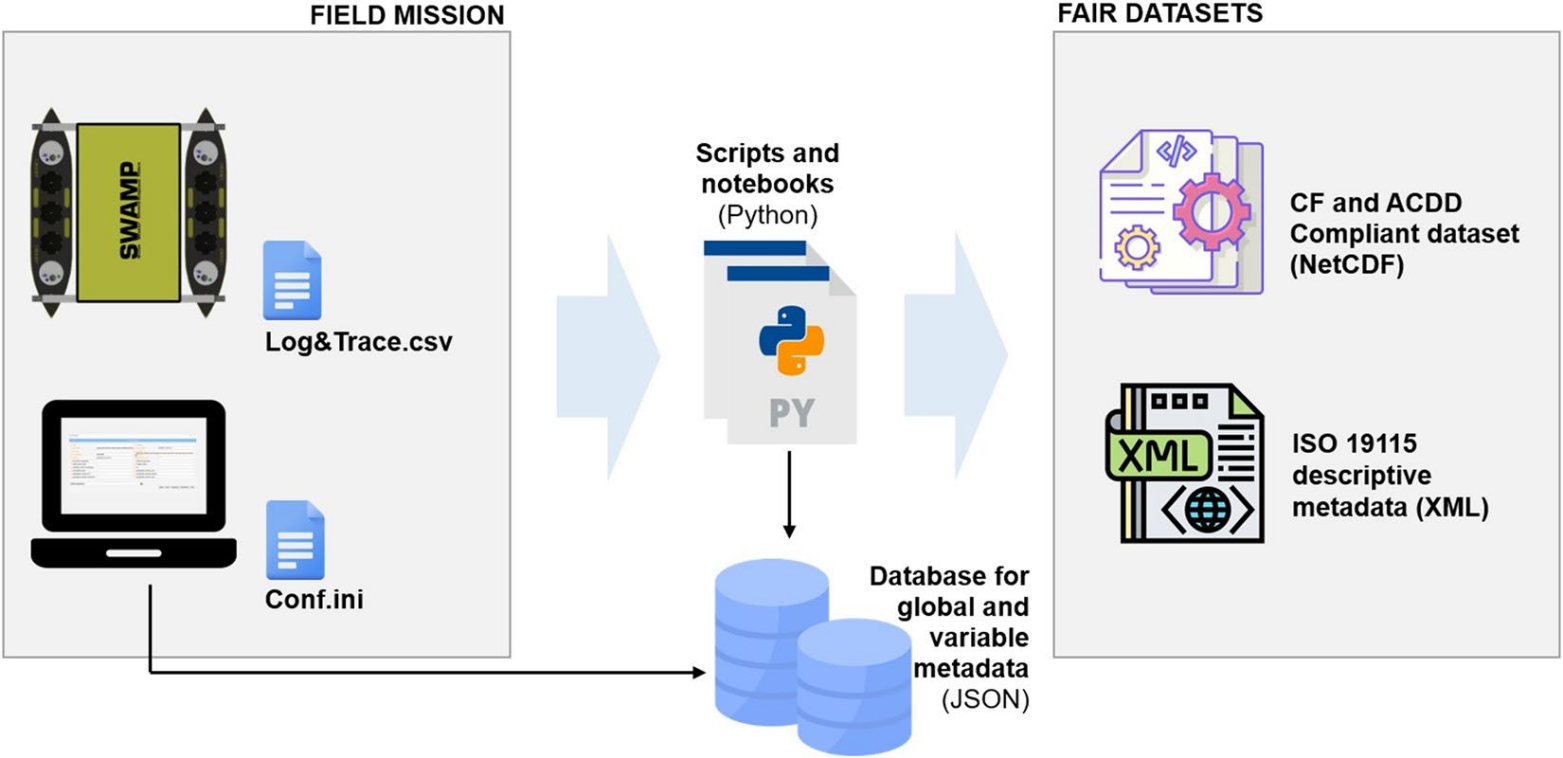
Schema for the automation and application of the method.

The proposed FOSS consists of a set of python scripts, Jupyter^32^ notebooks and modules to provide a metadata infrastructure, described in details in the method section. Applying the FOSS pipeline, hence the metadata infrastructure, during a field mission, results in the generation of a FAIR dataset, stemming from the telemetry of a robotic platform, namely SWAMP (Shallow Water Autonomous Multipurpose Platform)^33^. SWAMP is a catamaran-shaped vehicle with double-ended hulls that can host a number of different sensors on its deck. During the considered data campaign, SWAMP automatically performed a number of standardised maneuvers^34^ by following the International Towing Tank Conference (ITTC) criteria^35^. Fieldwork and expeditions in marine robotics^36^ are typically characterised by both a large number of daily planned activities and unexpected events. Therefore, it is unrealistic to rely on the operators to manually create a FAIR dataset during such missions. The discussed FOSS supports the automation of the process by attaching a minimum set of global and variable metadata after the data acquisition with minimal intervention on the operator’s behalf. Figure 1 schematises the proposed approach. The two files on the left represent the inputs to the FOSS, they contain respectively the telemetry of the marine platform and a configuration file. In this phase, a dedicate python module is used to connect to the database which contains global and variable metadata, to retrieve all the metadata information. We provide our data in the form of NetCDFs, which is a widely used format that enables the creation, access, and sharing of labeled and array-oriented data^20,21^. In addition to the NetCDF, the software pipeline also outputs an eXtensible Markup Language (XML) ISO 19115-compliant file, containing the descriptive metadata. The two input files are:

1. *Log&Trace.csv*: the vehicle’s telemetry data, in the form of a raw log table, provided by SWAMP
2. *Conf.ini*: the descriptive metadata, in the form of a configuration file, generated by the Human-Computer Interface (HCI) used to remotely control the vehicle

**Fig. 2:**
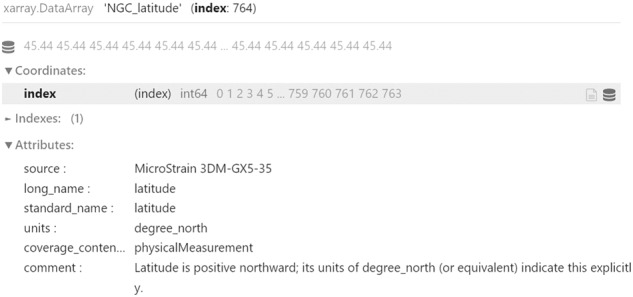
How the NGC_latitude variable appears in the NetCDF file, opened in Python with xarray. The instrument name is extracted from the variable and attached as an attribute.

**Fig. 3:**
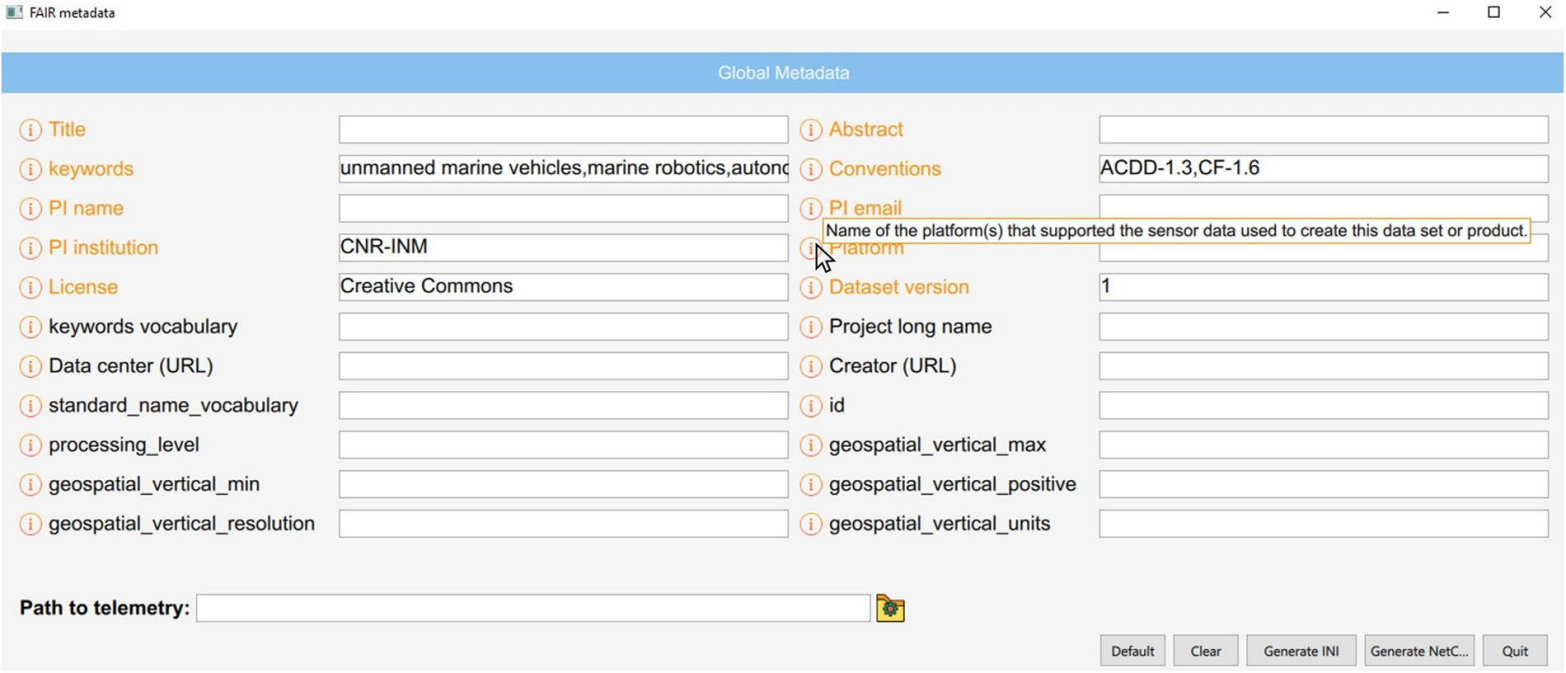
Interface view to add the global metadata. Mandatory values are in orange. All global metadata that can be automatically generated from the dataset are not shown in the view. It is possible to hover over the information icon to read the description of each metadata. The buttons on the bottom right can be used to add default values, to clear, and to generate the files.

**Fig. 4:**
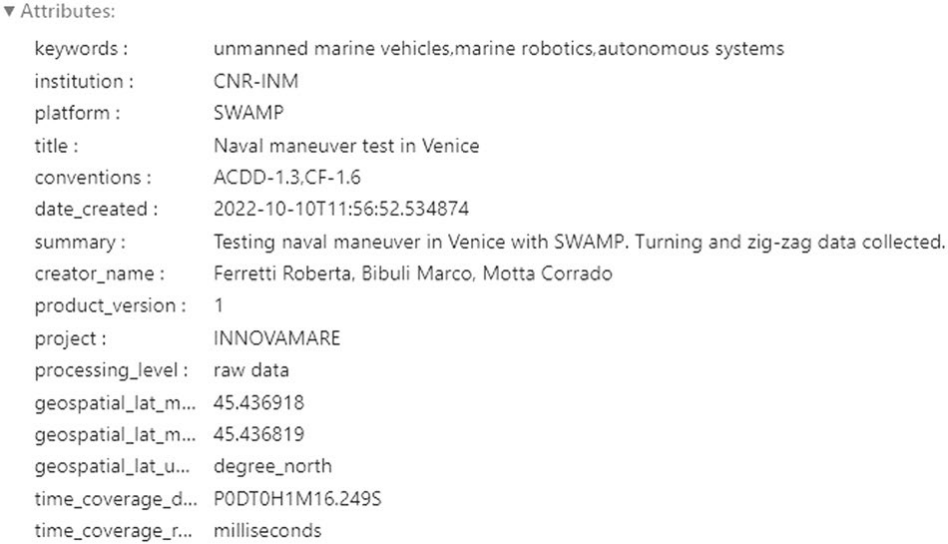
How the global metadata appears in the NetCDF file, opened in Python with xarray (only a subset is shown here).

The telemetry file is a text file storing the values of each variable in a dedicated column. For each column, we set a 2-level name, followed by all the values. The first level name is the custom name given to a specific variable (what we used to do already), whilst the second level name is the *long_name*, which indicates the standard variable name it refers to. Since the *long_name* is also the unique ID of the variable database, such information can be used by the scripts to retrieve from the database all the attributes of the pointed variable. It is fundamental to use a 2-level name for the columns, as it is very common to have multiple measurements for the same type of data. For example, SWAMP contains more than one Global Navigation Satellite System (GNSS) on board. One is part of the Navigation, Guidance and Control (NGC) unit, whilst the other is contained in the independent propulsion modules called *minions* and located in the hulls. During operations, the minions are identified with their positions: Front-Left (FL), Front-Right (FR), Rear-Left (RL), and Rear-Right (RR). Therefore, multiple latitude and longitude measurements will be collected during any SWAMP field deployment. Furthermore, to guarantee the replicability of the datasets, it is important to know the specific instruments used to obtain each individual measurement, or the particular algorithms used to process the data. By knowing them, it is also possible to find out the accuracy of each collected dataset. For these reasons, we include such information, when available, to the first level name, within square brackets, used as delimiters. Table 1 shows the 2-level names in the first row and the different instruments used, as should be reported in the log file. The script uses the first name to set the variable name on the NetCDF file and the second name as the ID of the database to retrieve all the attributes (e.g., unit, coverage_content_type, description, etc.) in order to append them to the NetCDF variable just created. Also the instrument name, if present, is extracted and removed from the variable name and saved as a variable metadata, under the CF attribute named *source*. When the NetCDF file is opened in Python, using the module *xarray*, the corresponding variable is shown as reported in Fig. 2. For the descriptive metadata, the vehicle’s interface provides a specific panel view to generate the input configuration file, as shown in Fig. 3. Such a view is directly connected to the database and gets automatically populated with the latest version of the global metadata and their attributes. It is therefore sufficient to add or edit an entry to the database and commit it to remote, to see it appearing in the operator’s HCI view as well. Each operator can specify new default values locally, in addition to the general ones provided by the database. In this fashion, most of the fields in the view can be filled with a single click. Once the mandatory fields are filled, the configuration file can be generated. Such a file contains a set of key-value entries, where the key is the name found in the Attribute Convention for Data Discovery (*ACDD*)^37^, the unique ID of the database. Ultimately, the scripts use the file to append the metadata to the NetCDF. Furthermore, it connects to the database to retrieve all metadata that should be automatically calculated (i.e., when the *auto* attribute is set to “True”) and generate them as well. For example, the *time_coverage_duration* can be calculated from the *date* and *time* standard variables of the vehicle’s log file, by following ISO 8601-1:2019^38^ proposed format^39^. Figure 4 shows how some of the generated global metadata appears on *xarray*.

**Table 1:** Example of 2-level names in SWAMP telemetry, followed by values.

NGC_latitude[Micro Strain 3DM-GX5-35]	NGC_longitude[Micro Strain 3DM-GX5-35]	FR_latitude[GPS U-blox Neo-M9N]	FR_longitude[GPS U-blox Neo-M9N]	[..]
*latitude*	*longitude*	*latitude*	*longitude*	
45.438759	12.327145	45.515624	12.419372	
45.438760	12.327148	45.515635	12.419332	[..]
45.438750	12.327103	45.515690	12.419345	

In this case, the first level contains the instrument name between square brackets.

## Discussion

Coupled environmental and robotics datasets, collected through innovative marine platforms, are fundamental to enable cross-analysis between the measurements, e.g., to decipher the environmental data based on the platform behaviour, to seal the best interpretation of marine telemetry and to generally increase the transparency of the performed field and model experiments. As full reproducibility of the results is often not possible due to the nature of the domain and the type of field missions, the proposed framework focuses on allowing the replication or the re-use of the data and on optimising the sharing of the obtained research results, in line with the EU research and innovation strategies^40^. In fact, rendering these unique datasets FAIR, enables interoperability and enhances their visibility. The resulting datasets are within reach of the wider scientific community. The framework presented here can be applied in simulations, where reproducibility is more likely. This could be part of a future work, for instance employing the MARUS simulator^41^ (https://marusimulator.github.io/).

The Free and Open Source Software described here, implements the best practices already in use in Earth Sciences and it is the ideal first step to create a “FAIR by default” dataset during the conclusion of a marine expedition, which involves emerging technology^42^. The automation provided by the scripts, facilitates and increases the creation of datasets equipped with the appropriate metadata, which in turn, eases the correct storage and publication of data and then eventually enables the creation of consistent time series, which are fundamental to study the evolution of natural phenomena taking place, in this instance, in the ocean. Furthermore, the work presented in this manuscript is a first fundamental step towards a deeper interdisciplinary discussion around a continuously evolving technological and observational system.

In this manuscript, we use SWAMP as an exemplary rising robotic platform, not commercialised. SWAMP is, in fact, an autonomous platform, highly modular and reconfigurable, hence capable of different sensing modalities. Its unconventional configuration renders the standardisation of the logged data more complex when compared to commercialised platforms. Other autonomous vehicles, such as gliders, are part of dedicated programs, are commercially available and their data logging is tailored for ocean observations. The framework described in this manuscript offers a coupled dataset, reporting the data describing the performance of the observing platform and the environmental data gathered during the same field campaign. A shared dedicated vocabulary in support of a FAIR coupled datasets is novel and fundamental to accelerate raising robotic platforms into the ocean observing system. For instance, it is useful to log variables related to the functioning of the control algorithms applied to sustain marine robotic research. Gliders are among established autonomous vehicles which benefit from bespoke data and metadata documentation by the OceanGliders group^43^ - formerly Everyone’s Gliding Observatories (EGO). The Data Management Team of EGO provides guidelines to format a NetCDF file for distributing glider data^44^ and metadata^43^ in a EGO-compliant manner^45^. They also provide a NERC-compliant vocabulary containing standard names for glider measurements. Therefore, the EGO community promotes glider technology and focuses on homogenising^44^ and standardising data collected by ocean gliders, hence greatly improving glider data sharing and scientific and international collaboration. The objective of our study is to provide a general-purpose framework, and its related code, to generate FAIR datasets from the collected data, in an automated way. Such a framework, is dedicated to emerging robotic platforms, not yet commercialised, but apt to perform unconventional measurements, at times in remote areas of the ocean^36,46^, thus operating in the observational gap left by established platforms. The software, proposed in this manuscript, is simple and open to guarantee prompt and effortless adaptation in the different operational and sensing modes that characterise emerging platforms.

## Methods

The proposed method can be split into two parts.

The first one concerns a review of the existing conventions that provide standardised metadata and their possible adaptation to marine robotics. Our objective is not to create yet another standard or data format, but to offer a set of simple tools that draw from what has already been developed for other scientific contexts. As already mentioned, the output file format is NetCDF. It allows the attachment of metadata information on both a global and variable level, which renders the dataset self-describing.

The second part is to build an infrastructure to access such metadata automatically, in order to create a “FAIR by default” dataset. In fact, one of the goals of this work is to generate FAIR robotic and scientific datasets at the end of every field mission. Such infrastructure is set to be as comprehensive as possible and can be adopted in other scientific contexts. In order to build our infrastructure, the fundamental components that we need to include to fully describe a dataset are the following:

- Descriptive (or global) Metadata
- Variables Standard Names
- Use (or variable) Metadata

A FAIR dataset shall be findable and accessible. To enable that, the data must be enriched with a set of “global” metadata typically referred to as descriptive metadata. Usually, such metadata are domain agnostic, many standards exist and are used and shared between different fields. For example, ISO 19115^11^ provides a schema to describe any kind of geographic information^47^. Descriptive Metadata answer questions such as the spatial and temporal dimension of the collected data as well as its identification, scientific summary, and its license.

Therefore, we started to define a minimum set of descriptive metadata for our datasets. An extract of such selection is reported in Table 2. Each entry comes with a number of attributes, e.g., the name, the description, whether the metadata can be automatically generated from the dataset, the default value, and whether is mandatory or optional to include. However, the most important field is the standard name (here reported as *ACDD*), because it has to come from a shared and controlled vocabulary, where the standard names are recognised worldwide and allow us to find automatically the datasets of interest.

**Table 2:** Defining a minimum set of global metadata to be associated with a given dataset.

Name	Description	ACDD	Auto
Title	A brief title for the dataset	title	no
Abstract	A short summary for dataset, the content and potential linkages etc.	summary	no
keywords	A comma separated list of key words and phrases	keywords	no
Conventions	A comma-separated list of the conventions followed by the dataset.	conventions	no
keywords vocabulary	Guideline for the words/phrases in your “keywords” attribute, if any	keywords_vocabulary	no
PI name	Name of the PI	creator_name	no
PI email	Email to the PI	creator_email	no
PI institution	Affiliation of the PI	institution	no
Dataset start time	ISO8601 reference for the dataset	time_coverage_start	yes
Dataset end time	ISO8601 reference for the dataset	time_coverage_end	yes
Dataset northernmost latitude	Geographical northernmost position of the dataset	geospatial_lat_max	yes
Dataset southernmost latitude	Geographical southernmost position of the dataset	geospatial_lat_min	yes
Dataset latitude units	Further refinement of the box	geospatial_lat_units	yes
Dataset easternmost longitude	Geographical easternmost position of the dataset.	geospatial_lon_min	yes
Dataset westernmost longitude	Geographical westernmost position of the dataset.	geospatial_lon_max	yes
Dataset longitude units	Further refinement of the box	geospatial_lon_units	yes
date created	The date on which the data was created.	date_created	yes
Platform	Name of the platform(s) that supported the sensor data.	platform	no
Project long name	The scientific project that produced the data.	project	no
License	Describe the restrictions to data access and distribution	license	no
Dataset version	Version identifier of the data file or product.	product_version	no
Data center (URL)	URL to the data center hosting the data	publisher_url	no
Creator (URL)	URL to creator or to information	creator_url	no
standard_name_vocabulary	The name of the controlled vocabulary for the variable standard names	standard_name_vocabulary	no
time_coverage_duration	Describes the duration of the dataset. Use ISO 8601:2019 format	time_coverage_duration	yes
time_coverage_resolution	Describes the time period between each value. Use ISO 8601:2019 format	time_coverage_resolution	yes
id	An identifier for the dataset, it can be the DOI as well	id	no
processing_level	A textual description of the processing level of the data	processing_level	no

Note that some attributes are omitted here, such as the default value and whether in the proposed FOSS it is considered mandatory or optional.

To define such a minimum set of global metadata, we reviewed the descriptive information that is typically asked in the marine robotic field and, in general, in the Earth Sciences domain. The SIOS (Svalbard Integrated Arctic Earth Observing System)^9,10^ and the Ocean Geospatial Consortium Catalog Service for the Web (OGC CSW)^47^ were the starting point. Once that we identified the metadata of interests, we aligned to the standard schema proposed by ISO 19115^11^. Finally, since our goal is to create NetCDF FAIR-compliant datasets, we converted the ISO 19115^11^ standard names, which are provided in the form of XML elements, to human-readable global metadata. To do that we used the ACDD. This convention contains the list of recommended global metadata for describing a NetCDF dataset and provides the mapping with the ISO 19115^11^ standard schema, which was used in this context. The final minimum set of global metadata is stored in a database-like format and is available in the dedicated GitHub space^30^. The unique field for each entry is named *ACDD*, it contains the standard name and is used as the ID of the database.

Once the dataset has all the ingredients to be discoverable, the second major issue to consider is interoperability. As a matter of fact, it is not enough to guarantee an automated discovery, access, and download of a dataset. It is equally, if not more important to enable the automated processing of the data contained in the dataset as well. A typical use case is the development of a script that downloads different datasets and analyses the data that are contained in them, for example by merging or comparing their measurements. In the marine robotic field, if such datasets are produced by different researchers, or even different research groups or institutions, there is a high probability that the variable names used to describe the same measurements are chosen independently, hence they do not match. This makes the automated analysis cumbersome, if not impossible, and increases exponentially the time needed to align the input data.

As a consequence, it is necessary to establish standard names also for the variables contained in the dataset. Currently, many EOV (Essential Ocean Variables)^48^ do have a correspondent standard name in a shared and controlled vocabulary, which enables interoperability. One of the most common conventions is the Climate and Forecast (CF) Metadata Convention^19^, which gathers the standard names that can be attached as a variable attribute in a NetCDF file (the attribute field is named *standard_name* by the CF convention itself). In this way, the operator can still set a custom name to a variable and then include the correspondent standard name as an attribute. However, for the robotic variables, i.e., variables describing the performance of the robotic platform, there is no such agreement and it is still a challenge to find standard names shared by the community.

Similarly to what was done with the global metadata, we started to define standard names for the robotic variables found in the telemetry of our robotic vehicles and to store them in a light database to quickly access them when needed. When possible, we used existing standard names for the variables, e.g., for common measurements such as yaw, pitch, and roll we used the CF names *platform_yaw*, *platform_pitch* and *platform_roll*. For all the other cases, where we could not find a name from a controlled vocabulary, we proposed a new standard name. We tried to cohere as much as possible with the terminology commonly used in the robotic field, by following Fossen’s guidelines. As shown in Table 3, for each log name, we filled both the field *standard_name* and the field *long_name* when we found a standard name from a controlled vocabulary such as CF, e.g. for *platform_yaw*. On the other hand, we filled only the attribute *long_name* when we proposed a new name, e.g., in the case of *platform_heave_acceleration_down*. In this way, the attribute *long_name* always contains a unique value and it can be used as the ID for the database. The complete, but preliminary list can be found in the dedicated GitHub space^30^.

**Table 3:** Mapping Standard Names with Log Names, exemplary table.

Log Name	Comment	Long Name	Standard Name	Unit	coverage_content_type
date	Date in format…	date			physicalMeasurement
time	Time in format…	time	time	s	physicalMeasurement
latitude	Latitude is…	latitude	latitude	degree_north	physicalMeasurement
longitude	Longitude is…	longitude	longitude	degree_east	physicalMeasurement
xgps	x indicates…	projection_x_coordinate	projection_x_coordinate	m	auxiliaryInformation
ygps	y indicates…	projection_y_coordinate	projection_y_coordinate	m	auxiliaryInformation
roll	Roll rotation…	platform_roll	platform_roll	degree	physicalMeasurement
pitch	Pitch rotation…	platform_pitch	platform_pitch	degree	physicalMeasurement
yaw	Yaw is a…	platform_yaw	platform_yaw	degree	physicalMeasurement
heave_acceleration	Heave…	platform_heave_acceleration_down		m s-2	auxiliaryInformation
lcCtdDepth	Depth is…	depth	depth	m	physicalMeasurement
lcCtdTemperature	Sea water…	sea_water_temperature	sea_water_temperature	degree_C	physicalMeasurement
lcCtdConductivity	Conductivity…	sea_water_electrical_conductivity	sea_water_electrical_conductivity	S m-1	physicalMeasurement

Log names are the names that every lab/mission/robot uses to log the variable in the raw format in a log file. The Long Name and the Standard Name should correspond; the standard name, when present, is the name found in a shared vocabulary. In this first version, only CF convention is considered.

Finally, for each log variable, besides the *standard_name* and *long_name* attributes, it is important to set other attributes that help the user or the machine to understand the characteristics of each measurement. These additional attributes include, for example, the unit of measurement, the fill value in case of missing data, or whether the log variable refers to a direct (physical) measurement or not. Also, such attributes shall have a standardised name to be processed automatically. We referred to them as variable metadata. Table 4 summarises the most important attributes, which can be attached to each variable in a NetCDF file. Right now, we include as mandatory only the ones indicated with “M” in the last column. The other fields are optional, but highly recommended. The *source* attribute is the only one that is not persistently assigned to a standard variable, but depends on the instruments used on a specific field mission or on the algorithms employed to pre-process the output data. Therefore, such information is attached to the custom variable name. The two databases generated, one containing the global metadata and one containing the variable standard names with their attributes (variable metadata), are living entities and are constantly updated. They are provided in the form of Lightweight JSON-based databases. They can be accessed or modified by adding, removing, or updating one or more entries with a simple Python module named “metadataDB” which is provided as part of the FOSS. The module is a customised wrapper of the public module named PysonDB-V2 and connects directly with the databases. An additional notebook named “database.ipynb” explains the module and how to access the database. The notebook, the module, and the JSON database files are available on GitHub^30^. These databases and the module to access them represent the simplest form of the proposed infrastructure and enable us to produce datasets composed by a single NetCDF file containing all global metadata, all variable metadata (attributes), and all the actual measurements, in an automated fashion.

**Table 4:** Attributes table, example.

Attribute	Convention	Description	M-O-NI
units	NUG/ACDD	A character string that specifies the units used for the variable’s data	O
long_name	NUG/ACDD	A long descriptive name. Used to define standard names.	M
_FillValue	NUG	To specify the fill value used to pre-fill disk space allocated to the variable.	O
_NoFill	NUG	Interpreted by the ncgen utility.	NI
missing_value	NUG	A scalar or vector containing values indicating missing data	NI
valid_min	NUG	A scalar specifying the minimum valid value for this variable.	O
valid_max	NUG	A scalar specifying the maximum valid value for this variable	O
valid_range	NUG	A vector of two numbers specifying the minimum and maximum valid values for this variable.	O
scale_factor	NUG	If present, the data shall be multiplied by this factor after the data are read by the application.	O
add_offset	NUG	If present, this number is to be added to the data.	O
Coordinates	NUG/CF	Identifies auxiliary coordinate variables	O
C_format	NUG	A character array to inform C application on the format to be used.	O
standard_name	CF/ACDD	Standard name following CF convention	O
coverage_content_type	ACDD	An ISO 19115-1 code to indicate the source of the data.	O
source	CF	Method of production of the original data.	O
comment	CF	Miscellaneous information about the data or methods used to produce it.	M
actual_range	CF	The smallest and the largest valid non-missing values occurring in the variable.	O

The list of attributes follows the indications of the Climate and Forecast Convention (CF), the NetCDF Users Guide Convention (NUG), and the Attribute Convention for Data Discovery (ACDD). The last column indicates the decision taken concerning the attributes in our dataset. M-O-NI stands for Mandatory, Optional, Not Included.

## Data Availability

An example of practical application of the proposed method on real data acquired during field tests to obtain FAIR robotic dataset is available on Zenodo repository^31^ at 10.5281/zenodo.7825000. The data record is composed of two files referring to the same dataset: the .csv file is the raw format that was acquired by the ASV robotic platform SWAMP during field test. The .nc file contains the same data, but in a standard format and with global and variable metadata generated using the standardization workflow, based on FAIR Principles, described in this paper, which uses controlled and standard vocabularies (ACDD and standard CF). The data refer to the execution of zig-zag manoeuvres of the ASV following the ITTC standards for ship manoeuvrability, adapted to the specific case of innovative surface robotic platform^34^. The shared dataset demonstrates the practical applicability of the proposed framework, hence adding value to our study. This is an example that can be extended to other datasets acquired with emerging surface robotic platforms in different contexts.

## Code availability

Scripts, notebooks and modules to generate metadata in several formats following FAIR principles for marine robotic data is available on GitHub (https://github.com/CorradoMotta/FAIR-Data-in-Marine-Robotics), under the GNU General Public License v3.0. The dedicated GitHub page^30^ of the project supports the understanding and usage of the codes.

## References

[1] Aracri S (2021). Soft robots for ocean exploration and offshore operations: a perspective. Soft Robotics.

[2] Piermattei V (2018). Cost-effective technologies to study the Arctic Ocean environment. Sensors.

[3] Bernardi M (2022). AURORA, a multi-sensor dataset for robotic ocean exploration. International Journal of Robotics Research.

[4] GOOS. Global Ocean Observing System. https://www.goosocean.org/ (2021).

[5] Aracri, S. *et al*. Open science in marine robotics. In *International Conference on Open Data (ICOD 2022): Book of abstracts*, 96–100, 10.5281/zenodo.8071065 (2023).

[6] Wilkinson MD (2016). The FAIR guiding principles for scientific data management and stewardship. Scientific Data.

[7] NASA. Global Change Master Directory - Directory Interchange Format (GCMD DIF). https://www.earthdata.nasa.gov/esdis/esco/standards-and-practices/directory-interchange-format-dif-standard (2023).

[8] SIOS. Svalbard Integrated Arctic Earth Observing System. https://sios-svalbard.org/ (2021).

[9] SIOS. Technical documentation guidance for data centres contributing to SDMS. https://www.sios-svalbard.org/sites/sios-svalbard.org/files/common/SDMS_Interoperability_Guidelines.pdf (2020).

[10] Ignatiuk, D. *et al*. SIOS data management system: distributed data system for Earth system science. In *EGU General Assembly*, vol. 19–30 April, EGU21–15205, 10.5194/egusphere-egu21-15205 (2021).

[11] International Organization for Standardization. ISO 19115-1:2014 geographic information – metadata – part 1: fundamentals. https://www.iso.org/standard/53798.html (2014).

[12] Waldmann, C. *et al*. About the value of standards for ocean technology. In *OCEANS 2021: San Diego – Porto*, 1–5, 10.23919/OCEANS44145.2021.9705984 (2021).

[13] NORSOK Standard. U-102 Remotely operated vehicle (ROV) services. https://online.standard.no/norsok-u-102-2020 (2020).

[14] DNV-GL. Rules for classification - underwater technology part 5 types of UWT systems, chapter 7 remotely operated vehicles. https://www.dnv.com/ (2015).

[15] Gabl R (2021). Hydrodynamic loads on a restrained ROV under waves and current. Ocean Engineering.

[16] Walker KL (2021). Experimental validation of wave induced disturbances for predictive station keeping of a remotely operated vehicle. IEEE Robotics and Automation Letters.

[17] NATO, STANDARD ANEP-87. Digital underwater signalling standard for network node discovery & interoperability. Edition A Version 1. https://nso.nato.int/nso/nsdd/main/standards?search=ANEP-87 (2017).

[18] DNV-GL. Rules for classification - Underwater Technology Part 5 Types of UWT systems, Chapter 8 Autonomous underwater vehicles. https://www.dnv.com/ (2015).

[19] Eaton, B. *et al*. NetCDF Climate and Forecast (CF) metadata conventions. http://cfconventions.org/Data/cf-conventions/cf-conventions-1.10/cf-conventions.pdf (2022).

[20] Brown SA, Folk M, Goucher G, Rew R (1993). Software for portable scientific data management. Computers in Physics.

[21] Rew R, Davis G (1990). NetCDF: an interface for scientific data access. IEEE Computer Graphics and Applications.

[22] Flanders Marine Institute. Marine regions. Managed by Flanders Marine Institute. https://www.marineregions.org/ (2018).

[23] National Oceanographic Center. The NERC Vocabulary Server (NVS). https://vocab.nerc.ac.uk/ (2023).

[24] Freeland, H. *et al*. ARGO - a decade of progress. In *Proceedings of OceanObs'09: Sustained Ocean Observations and Information for Society*, **Vol. 2**, 357–370 (European Space Agency, 2010).

[25] Schoening T (2022). Making marine image data FAIR. Scientific Data.

[26] Schoening T (2022). Publisher correction: making marine image data FAIR. Scientific Data.

[27] Fossen, T. I. *Guidance and control of ocean vehicles* (Wiley, 1994).

[28] DOI Foundation. The DOI® Handbook. https://www.doi.org/the-identifier/resources/handbook (2019).

[29] Stocker, M. *et al*. Persistent identification of instrument. *Data Science Journal* 19 (2020).

[30] Motta, C., Ferretti, R. & Aracri, S. FAIR data in marine robotics. Zenodo. v0.1.0-alpha. 10.5281/zenodo.8256384, https://corradomotta.github.io/FAIR-Data-in-Marine-Robotics/html/index.html (2023).

[31] Ferretti R, Motta C, Bibuli M (2023). Zenodo.

[32] Team, J. Project Jupyter. https://jupyter.org/ (2023).

[33] Odetti A, Bruzzone G, Altosole M, Viviani M, Caccia M (2020). SWAMP, an autonomous surface vehicle expressly designed for extremely shallow waters. Ocean Engineering.

[34] Ferretti, R. *et al*. Procedures for maneuverability characterization: from ships to marine robots. In *Computer Applications and Information Technology in the Maritime Industries, COMPIT'23*, http://data.hiper-conf.info/compit2023_drubeck.pdf (2023).

[35] Quality Systems Group of the 29th International Towing Tank Conference. ITTC recommended procedures and guidelines. https://ittc.info/media/9876/0_0.pdf (2021).

[36] Bruzzone, G. *et al*. Multi-sensor 3D mapping of Tethys Bay (Ross Sea – Antarctica) with PROTEUS, an innovative, highly reconfigurable and versatile unmanned marine vehicle. In *EGU General Assembly 2023*, EGU23–12041, 10.5194/egusphere-egu23-12041 (2023).

[37] (ESIP), E. S. I. P. Attribute Convention for Data Discovery - ACDD. https://wiki.esipfed.org/Attribute_Convention_for_Data_Discovery_1-3 (2022).

[38] International Organization for Standardization. ISO 8601-1:2019 date and time – representations for information interchange – part 1: basic rules. https://www.iso.org/standard/70907.html (2019).

[39] Intergovernmental Oceanographic Commission of UNESCO. Ocean Data Standards, Vol.2: Recommendation to adopt ISO 8601:2004 as the standard for the representation of dates and times in oceanographic data exchange. https://repository.oceanbestpractices.org/bitstream/handle/11329/217/54_2.pdf?sequence=1 isAllowed = y (2011).

[40] Lee, B. *et al*. Reproducibility of scientific results in the EU: scoping report. https://www.ouvrirlascience.fr/wp-content/uploads/2020/12/Reproducibility-of-scientific-results-in-the-EU.pdf (2020).

[41] Lončar, I. *et al*. MARUS - a marine robotics simulator. In *OCEANS 2022, Hampton Roads*, 1–7 (2022).

[42] EuroGOOS Office, EuroGOOS Technology and Planning Working Group Chairs & EOOS Technology Forum Foresight Workshop Organising Committee. EOOS technology forum report 2022. Thinking ahead: the technology of the science we need for the ocean we want. https://www.eoos-ocean.eu/wp-content/uploads/2022/08/EOOS-Tech-Forum-report-2022.pdf (2022).

[43] OceanGliders. Ocean gliders: data and metadata from Global Data Assembly Centre. 10.17882/56509 (2023).

[44] EGO gliders data management team. EGO gliders data processing chain. 10.17882/45402 (2023).

[45] Thierry, C., Claire, G., Jean-Philippe, R., Justin J. H. B. & Bartolome, G. EGO gliders NetCDF format reference manual. 10.13155/34980 (2023).

[46] Bruzzone, G., Odetti, A., Caccia, M. & Ferretti, R. Monitoring of sea-ice-atmosphere interface in the proximity of Arctic tidewater glaciers: the contribution of marine robotics. *Remote Sensing* **12**, 10.3390/rs12111707 (2020).

[47] Ocean Geospatial Consortium Catalog Service for the Web - OGC CSW. https://www.ogc.org/ (2023).

[48] Lindstrom, E., Gunn, J., Fischer, A., McCurdy, A. & Glover, L. A framework for ocean observing. https://unesdoc.unesco.org/ark:/48223/pf0000211260 (2012).

